# The Role of Rho Kinase in Sex-Dependent Vascular Dysfunction in Type 1 Diabetes

**DOI:** 10.1155/2010/176361

**Published:** 2010-03-24

**Authors:** Daniel W. Nuno, Kathryn G. Lamping

**Affiliations:** ^1^The Department of Veterans Affairs Iowa City Health Care System, Roy J. and Lucille A. Carver College of Medicine, University of Iowa, IA 52246, USA; ^2^Department of Internal Medicine, Roy J. and Lucille A. Carver College of Medicine, University of Iowa, IA 52242, USA; ^3^Department of Pharmacology, Roy J. and Lucille A. Carver College of Medicine, University of Iowa, IA 52242, USA

## Abstract

We hypothesized that rho/rho kinase plays a role in sex differences in vascular dysfunction of diabetics. Contractions to serotonin were greater in isolated aortic rings from nondiabetic males versus females and increased further in streptozotocin-induced diabetic males but not females. The increased contractions to serotonin in males were reduced by inhibitors of rho kinase (fasudil, Y27632 and H1152) despite no change in expression of rhoA or rho kinase. Contractions to U46619 were not altered by fasudil or Y27632 or the presence of diabetes. In contrast to acute effects of fasudil, chronic treatment with fasudil increased contractions to serotonin in aorta from both non-diabetic and diabetic males. In summary, serotonin-induced contractions were increased in aorta from diabetic males but not females. Although administration of rho kinase inhibitors acutely decreased contractions to serotonin, long-term treatment with fasudil increased contractions. Long-term fasudil treatment may increase compensatory mechanisms to enhance vasoconstrictions.

## 1. Introduction

Typically women develop cardiovascular disease 10–15 years later than age-matched men; however, the benefits of female sex may not extend to diabetes. Epidemiological studies relating development of cardiovascular disease in diabetics with sex have not generated consistent results. While some studies have concluded that female sex and hormone replacement therapy reduce the risk of cardiovascular disease even in the presence of diabetes [1–3], others suggest that diabetes abolishes the benefits of female sex or even increases the risk of developing cardiovascular disease in women [4–10]. Studies in animal models of diabetes, in general, suggest a protective advantage or no effect of female sex [11–14]. Even in the absence of any protective effect of female sex, the underlying mechanisms responsible for alterations in vascular function may differ in male versus female diabetics. 

 The authors [15] and others [16] have demonstrated that sex differences in signaling pathways within vascular muscle contribute to differences in contractile function. Arteries from male mice contract to a greater magnitude to selected agonists compared to arteries from females [15, 17, 18]. This difference in contractile response was not related to the contribution of nitric oxide (NO) but rather due to agonist-specific activation of signaling pathways that regulate calcium sensitivity of contractile proteins. Activation of both rhoA and rho kinase which regulate myosin light chain phosphorylation was greater in arteries from males compared to females [15]. Rho/rho kinase signaling is increased in hypertension and atherosclerosis and may contribute to alterations in vascular function and heightened contractile states such as cerebral and coronary vasospasm [19]. In light of the sex difference in the activation of this pathway within blood vessels, we hypothesized that a sex-dependent difference in rho and rho kinase activation may contribute to differences in vascular dysfunction in male compared to female diabetics. We demonstrated in a model of Type 2 diabetes that serotonin-induced contractile responses were increased in aorta from both males and females but activation of rhoA and rho kinase was increased only in diabetic males and not females [20]. Thus, despite similar contractile dysfunction in arteries from male and female Type 2 diabetics, the dysfunction in diabetic males demonstrated greater dependence upon activation of rhoA and rho kinase. We concluded that alternative mechanisms regulating vasoconstriction may play a greater role in females.

 The goals of the present study were first, to determine whether sex differences in contractile function were observed in a model of Type 1 diabetes (streptozotocin-induced insulinopenia) and whether this was due to a difference in rho kinase signaling. We hypothesized that vascular dysfunction may be greater in arteries from Type 1 diabetic males compared to females due, in part, to a greater activation of rho/rho kinase signaling. The second goal was to determine whether chronic inhibition of rho kinase would prevent the development of vascular dysfunction in diabetic males. We hypothesized that if an increase in rho kinase activity contributed to the contractile dysfunction in diabetes then treatment with a rho kinase inhibitor may protect the arteries. To test these hypotheses, we compared reactivity of arteries from streptozotocin-induced diabetic male and female mice to nondiabetics. To determine the role of rhoA and rho kinase, we compared effects of specific rho kinase inhibitors on contractile responses in arteries from nondiabetic compared to diabetic males and females. Finally, we tested whether chronic administration of a rho kinase inhibitor to streptozotocin-induced diabetic mice would prevent development of vascular dysfunction.

## 2. Materials and Methods

### 2.1. Experimental Model

The animal protocol was reviewed and approved by the Animal Care and Use Committees at the Department of Veterans Affairs Iowa City Health Care System and the University of Iowa. All animal procedures complied with the “Guiding Principals for Research Involving Animals and Human Beings” of the American Physiological Society. Diabetes was induced in male and female C57BL6 mice 5-6 weeks of age by injection with streptozotocin (2-3 injections, 100–150 mg/kg ip) approximately one week apart. Mice were weighed and blood glucose was measured weekly. Diabetes was confirmed when blood glucose levels exceeded 300 mg/dl. 

### 2.2. Measurement of Vascular Reactivity

Studies were performed in aorta since it is the most commonly used mouse vascular model studied to date and allows us to compare vascular reactivity with protein expression and activity in the same vessel type. Responses of aorta were measured using previously published methods [15, 17, 20]. Nondiabetic and diabetic mice (12–16 weeks post first measurement of hyperglycemia) were anesthetized with pentobarbital (150 mg/kg ip), and the thoracic aorta was rapidly removed and placed in ice-cold Krebs buffer (mmol/L :  NaCl 118.3, KCl 4.7, CaCl_2_ 2.5, MgSO_4_ 1.2, KH_2_PO_4_ 1.2, NaHCO_3_ 25, and glucose 11). Aorta was cut into rings (3-4 mm in length) and mounted on wires connected to a force transducer in an organ bath filled with Krebs at 37°C aerated with 20% O_2_, 5% CO_2_, and balance N_2_. Tension was increased to 0.75 g over 60 minutes. To measure relaxation, rings were initially contracted with U46619 (10–20 nM) to maintain a stable contraction of 50%–60% of maximum before addition of acetylcholine (10 nM to 10 *μ*M) or sodium nitroprusside (10 nM to 10 *μ*M). Relaxation responses were expressed as percent decrease in tension from the initial contraction. Contractile responses to serotonin (10 nM to 1 *μ*M) or the thromboxane mimetic U46619 (10 nM to 1 *μ*M) were obtained by adding increasing concentrations to vessels under basal conditions. In general, 1-2 rings of aorta from each animal were used as controls and 1-2 were treated with inhibitors for 30 minutes prior to initiation of concentration response curves. A minimal contraction of 250 mg to KCl (75–100 mM) and greater than 50% relaxation to nitroprusside (10 *μ*M) was considered acceptable for inclusion in the study. In some groups contractions were compared before and after inhibition of rho kinase with fasudil (1-(5-Isoquinolinesulfonyl) homopiperazine hydrochloride, 10 *μ*M), Y27632 (trans-4-[(1R)-1-Aminoethyl]-N-4-pyridinylcyclohexanecarboxamide dihydrochloride, 1 *μ*M), or H1152 ((S)-(+)-2-Methyl-1-[(4-methyl-5-isoquinolinyl) sulfonyl]-hexahydro-1H-1,4-diazepine dihydrochloride, 1 *μ*M). Fasudil was used because of its clinical use in the treatment of cerebral vasospasm but it also inhibits protein kinase A (PKA), C (PKC), and G (PKG), myosin light chain kinase (MLCK), and CAM kinase II [21]. Y27632 which competes with ATP for binding to the catalytic domain of rho kinase is 100 times more specific for rho kinase compared to PKA, PKC, or MLCK [22, 23]. H1152 is a more specific and efficacious inhibitor of rho kinase than fasudil or Y27632 and a poor inhibitor of the serine/threonine kinases, PKA, PKC, and MLCK [24–26]. 

 Fasudil is approved for use in the treatment of cerebral vasospasm after subarachnoid hemorrhage in Japan and clinical trials suggest that it is beneficial in the treatment of angina [27]. To determine whether chronic inhibition of rho kinase with fasudil protects arteries of Type 1 diabetics from development of dysfunction, streptozotocin-treated male mice and age-matched nondiabetic mice were administered fasudil (100 mg/kg/day) in their drinking water for 12–15 weeks prior to study [28, 29]. Responses of isolated rings of aorta were compared to age-matched nontreated nondiabetics and diabetics as described above.

### 2.3. Western Blot Analysis

Aortas (3-4 aorta pooled per sample) from nondiabetic and diabetic mice were isolated and flash frozen in liquid nitrogen, minced, and sonicated in buffer containing 25 mM sucrose, 50 mM MOPS, 2 mM EDTA; 2 mM EGTA pH 7.4, Complete Protease Inhibitor (Roche Molecular Biochemicals), 50 mM NaF, 20 mM Na pyrophosphate, 1 mM p-Nitrophenyl phosphate, and 1 *μ*M Microcystin LR. The homogenate was centrifuged at 14,000 × g for 15 minutes at 4°C and protein concentrations were determined in the supernatant by the bicinchoninic acid method. Equal amounts of protein (30 or 50 *μ*g) were separated by SDS-PAGE gel electrophoresis. After blocking in milk, immunoblotting was performed using anti-rhoA (1 : 250, Santa Cruz), anti-rho kinase (ROCK1 or ROCK2, 1 : 500, BD Biosciences), or *α*-actin (1 : 500, Sigma) in 3% diluent followed by secondary antibodies conjugated with horseradish peroxidase. Immunoreactivity was visualized with enhanced chemiluminescence. Blots were digitized and normalized to actin for comparison (NIH Image).

### 2.4. Materials

All chemicals were purchased from Sigma-Aldrich unless otherwise noted. Y27632 and H1152 were purchased from Tocris Bioscience and U46619 from Biomol. Fasudil was purchased from Tocris and LC Laboratories.

### 2.5. Statistical Analysis

All data are presented as mean ± SEM. Responses of multiple rings from a given animal treated similarly were averaged and “*n*” represents numbers of mice per group. Concentration response curves were compared by repeated measures or a two- way analysis of variance followed by Bonferroni. Data from immunoblots were compared by Student *t* tests. Significance was defined at *P* < .05.

## 3. Results

### 3.1. Development of Hyperglycemia in Males and Females

Body weights and blood glucose levels for all groups are listed in Table 1. Blood glucose levels were similar for age-matched nondiabetic male and female mice. Following injection with streptozotocin, levels of blood glucose were more than doubled in both male and female mice (*P* < .05 versus nondiabetic). The increase in blood glucose levels was significantly greater in males compared to females (*P* < .05). Both nondiabetic and diabetic females weighed less than males and diabetic mice weighed less than nondiabetic mice (Table 1).

### 3.2. Sex-Dependent Differences in Relaxation Responses in Diabetes

**Figure 1:**
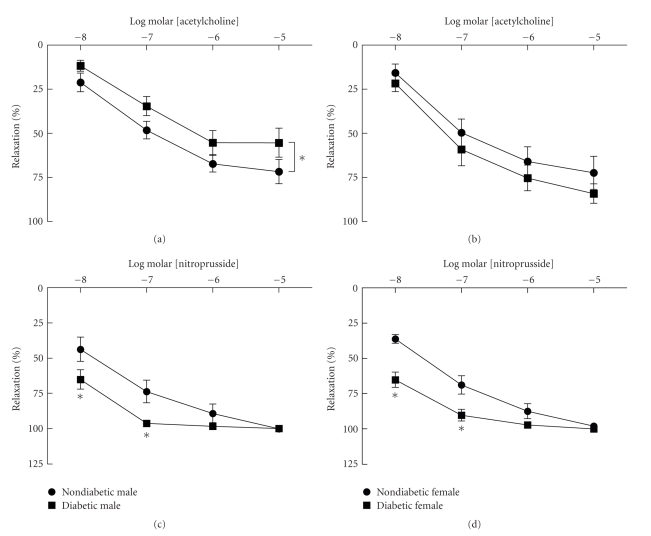
Relaxation of aorta from nondiabetic and diabetic males (left panels) and females (right panels) to acetylcholine ((a) and (b)) and nitroprusside ((c) and (d)). Relaxation to acetylcholine and nitroprusside was similar in nondiabetic males and females. Relaxation to acetylcholine was impaired in male but not female diabetics. In contrast, relaxation to nitroprusside was increased in both male and female diabetics (*n* = 8–14 per group, *P < .05 versus nondiabetic).

Similar to our previous findings [15], NO-mediated relaxation in response to acetylcholine and nitroprusside was similar in aorta from nondiabetic male and female mice (Figure 1). In aorta from diabetic males, there was a modest but significant reduction in relaxation to acetylcholine following development of hyperglycemia compared to nondiabetics (Figure 1(a)), however, differences in relaxation were not significant at any individual concentration of acetylcholine. In contrast, there was no change in relaxation in response to acetylcholine in aorta from diabetic females (Figure 1(b)). Relaxation in response to nitroprusside was increased at lower concentrations in arteries from both male and female diabetics compared to nondiabetics (Figures 1(c) and 1(d)). These results suggest that after 12–16 weeks of diabetes NO-mediated endothelium-dependent dilation is modestly impaired in males but not females. In contrast, relaxation of vascular muscle to exogenously administered NO released from nitroprusside is increased when relaxation to acetylcholine is either not altered or only minimally diminished.

### 3.3. Sex-Dependent Differences in Contractile Responses in Diabetes

**Figure 2:**
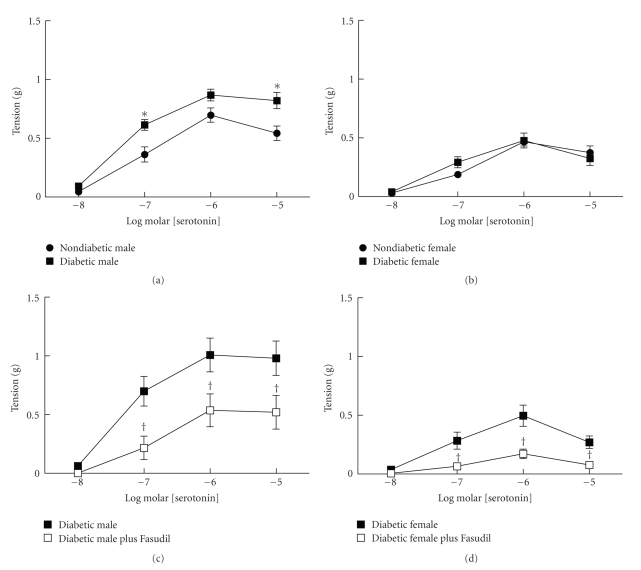
Contractions of aorta from nondiabetic and diabetic males and females to serotonin. Contractions to serotonin were greater in aorta from nondiabetic males (a) than females ((b) *n* = 13–15 per group, *P* < .05). Following development of diabetes, contractions to serotonin were increased in males ((a) *n* = 14) but not females ((b) *n* = 12, *P < .05 nondiabetic versus diabetic). In diabetic males and females inhibition of rho kinase with fasudil (10 *μ*M) reduced contractions to serotonin ((c) males, *n* = 5, (d) females, *n* = 8, ^†^ *P* < .05 diabetic versus diabetic plus fasudil).

In nondiabetic mice, contractions to serotonin were greater in aorta from males compared to females (Figures 2(a) and 2(b)) similar to our previous studies demonstrating sex-dependent differences in contractile responses to serotonin [15, 17]. Contractions of aorta from male mice were greater at 0.1 and 1 *μ*M serotonin (*P* < .05) compared to responses of aorta from females. In addition to a greater response to serotonin of aorta from nondiabetic males, contractions to serotonin were increased only in diabetic males and not diabetic females (Figures 2(a) and 2(b)). Maximal contractions of aorta from diabetic males to serotonin were almost two-fold greater than contractions of aorta from diabetic females. 

### 3.4. Role of Rho Kinase in Sex-Dependent Contractions in Type 1 Diabetics

Rho kinase plays a role in the increased contractile responses of arteries in other models of diabetes [20, 30, 31]. To examine the role of rho kinase in increased contractile responses to serotonin in streptozotocin-induced diabetes, we compared responses to serotonin before and after inhibition of rho kinase with fasudil in aorta from nondiabetic and diabetic males and females. Inhibition of rho kinase with fasudil (10 *μ*M) inhibited contractions of aorta from both diabetic males and females to serotonin by approximately 50% (Figures 2(c) and 2(d)). In the presence of fasudil, contractions of aorta from diabetic males to serotonin were significantly greater at 1 and 10 *μ*M compared to responses of arteries from diabetic females.

**Figure 3:**
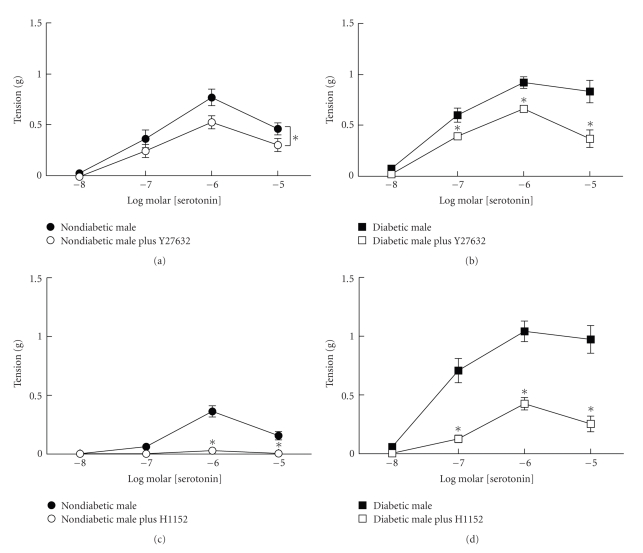
Effects of rho kinase inhibitors Y27632 (1 *μ*M (a) and (b)) and H1152 (1 *μ*M (c) and (d)) on contractions to serotonin in aorta from nondiabetic (left panels) and diabetic male mice (right panels). Inhibition of rho kinase with either Y27632 or H1152 decreased contractions of aorta from both nondiabetic and diabetic mice to serotonin (nondiabetics *n* = 4–6 per group, diabetics *n* = 8 per group, *P < .05 compared to paired untreated).

 Both Y27632 (1 *μ*M) and H1152 (1 *μ*M) inhibited contractions to serotonin in aorta from nondiabetic and diabetic male mice (Figure 3). In arteries from diabetic male mice, inhibition of rho kinase with Y27632 and H1152 reduced contractions to the levels in nondiabetic male mice. These results suggest that a major component of the enhanced contraction to serotonin in arteries from diabetic male mice was mediated by activation of rho kinase. 

 To determine whether the sex difference in contractile dysfunction in diabetics was agonist specific, we compared responses to thromboxane A_2_ mimetic U46619 in aorta from nondiabetic and diabetic mice. In nondiabetic mice, aorta from males contracted more than females but only at the lowest concentration of U46619 (10 nM, *P* < .05, data not shown). Maximal contractions were similar (0.1 *μ*M males 1.19 ± 0.09 g versus females 1.05 ± 0.06 g). In contrast to serotonin, contractions to U46619 were not increased in aorta from either diabetic males or females compared to nondiabetics (0.1 *μ*M :  males 1.31 ± 0.08 g and females 0.95 ± 0.07 g). Contractions to U46619 were not altered by fasudil or Y27632 but were decreased in aorta from both nondiabetics and diabetics by H1152 (data not shown). Thus, an increased contractile response was not a generalized phenomenon of diabetic vasculature but specific for serotonin in this model.

### 3.5. Expression of Rho Kinase and RhoA in Arteries from Diabetic Mice

**Figure 4:**
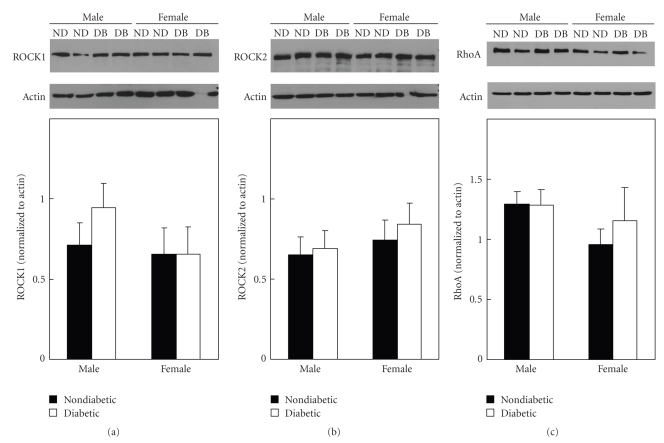
Representative immunoblots and mean levels of protein expression of ROCK1 (a), ROCK2 (b), and rhoA (c) in aorta from nondiabetic (ND) and diabetic (DB) males and females. Expression of rho kinase (ROCK1 and ROCK2) and rhoA was not different between males and females or nondiabetics and diabetics (mean ± SEM normalized to actin, *n* = 5-6 pooled samples each containing nondiabetic and diabetic males and females).

To determine whether the increase in contractions to serotonin of aorta from diabetic males and not females was due to a change in the expression of rho kinase, we compared expression of ROCK1 and ROCK2 in aorta from nondiabetic and diabetic male and female mice. Similar to our previous findings [15], expression of ROCK1 and ROCK2 was not different between nondiabetic males and females (Figures 4(a) and 4(b)). In addition, there was no change in expression or either rho kinase isoform in aorta of diabetics compared to nondiabetics (Figures 4(a) and 4(b)).

 Since activation of rho kinase is regulated, in part, by activation of the upstream rho GTPase, rhoA, we compared expression of rhoA in aorta from nondiabetic and diabetic males and females. Similar to our findings with rho kinase, there was no difference in expression of rhoA in nondiabetic males and females or between nondiabetics and diabetics (Figure 4(c)). Thus, the increased activation of the rho/rho kinase pathway in aorta from streptozotocin-induced diabetic mice was not due to a change in expression of either rhoA or rho kinase.

### 3.6. Effect of Chronic Treatment with Fasudil on Vascular Dysfunction in Diabetic Mice

**Figure 5:**
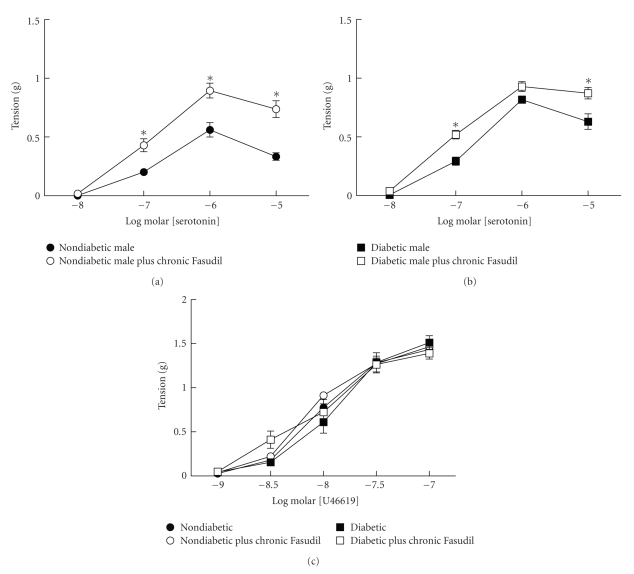
Effects of chronic treatment with fasudil (100 mg/kg/day, po) on contractions of aorta from nondiabetic and diabetic male mice to serotonin ((a) and (b), resp.). In contrast to effects of acute inhibition of rho kinase, chronic treatment with fasudil increased contractions of aorta from both nondiabetics and diabetics to serotonin (*P < .05 versus untreated). Chronic treatment with fasudil had no effect on contractions to U46619 in aorta from either nondiabetics or diabetics (c).

Since acute inhibition of rho kinase restored contractile responses of arteries from diabetic males to nondiabetic levels, we hypothesized that chronic inhibition of rho kinase would prevent development of vascular dysfunction in diabetics. Male mice were treated with fasudil for 12–16 weeks upon development of hyperglycemia (blood glucose over 300 mg/dl). Treatment with fasudil had no effect on body weight or level of hyperglycemia (Table 2). Measurements of vascular function at the end of the treatment period revealed surprising results. Similar to the previous results, contractions to serotonin were increased in aorta from diabetic males while contractions to U46619 were not different from nondiabetics (Figure 5). Interestingly, contractions to serotonin were increased in aorta from both nondiabetic and diabetic mice treated with fasudil compared to nontreated mice (Figures 5(a) and 5(b)). In fact, contractions to serotonin of aorta from nondiabetic mice treated with fasudil were similar to contractions of aorta from diabetic mice treated with fasudil. Contraction in response to U46619 was not changed in diabetics and was unaffected by treatment with fasudil in nondiabetics and diabetics (Figure 5(c)). Thus, in contrast to acute inhibition of rho kinase with fasudil, chronic treatment with fasudil increased contractions to serotonin of arteries from both nondiabetic and diabetic mice.

## 4. Discussion

There were several novel findings in the present study. First, development of vascular dysfunction in a model of Type 1 diabetes was dependent upon sex. Relaxation to acetylcholine was modestly impaired only in aorta from male diabetics and not females. Contraction to serotonin was augmented only in aorta from diabetic males and not females. This contrasts with the augmented relaxation to nitroprusside which was observed in arteries from both diabetic males and females. Second, the sex-dependent difference in contractions to serotonin may be related to an augmented activation of rho kinase; however, neither expression of rhoA nor rho kinase was increased in aorta of diabetic males compared to nondiabetics. Third, activation of rho kinase played a greater role in contractions to serotonin compared to U46619. This difference in the rho kinase component of the response may contribute to the greater contractions to serotonin and not U46619 in diabetic arteries. Fourth, despite a reduction in contractile response to serotonin in aorta from both nondiabetic and diabetic males following acute fasudil administration, chronic treatment with fasudil increased contractions to serotonin in aorta from both nondiabetic and diabetic mice. Fasudil treatment had no effect on contractions to U46619. These findings demonstrate that chronic treatment with fasudil did not prevent development of vascular dysfunction in Type 1 diabetics but actually enhanced contractions to serotonin. 

 The results of the present study, which demonstrate sex-dependent differences in contractions to serotonin of arteries from nondiabetic mice, are in agreement with our previous studies [15, 17]. In the present study, contractions to serotonin and not NO-mediated vasodilators were greater in arteries from males compared to females. Although numerous studies suggest that gonadal hormones regulate expression of nitric oxide synthase and release of NO, the authors and others have not detected differences in vascular responses to NO-dependent vasodilators in arteries from male compared to female mice [15, 32]. The reduced vasoconstriction to serotonin in arteries from female mice mediated by rho kinase [15] is in agreement with studies demonstrating estrogen-induced decreases in rho/rho kinase activity under basal conditions [16]. Although estrogen at supraphysiological levels decreases transcript for rho kinase in cultured cells [33], The authors and others have demonstrated a sex difference in rho kinase activity despite no difference in protein expression in arteries from male and female mice [15, 16]. Results of the present study confirmed our previous results that expression of rhoA and rho kinase was not different in aorta between nondiabetic C57BL/6 male and female mice [15]. This difference in contractions to serotonin of arteries from male compared to female mice has not been demonstrated in all mouse strains suggesting that genetic background influences the phenotype [20]. 

 In addition to differences in responses to serotonin depending on mouse strains, the model of diabetes also influences contractions of arteries to serotonin. The results of the previous and present studies suggest that the interaction between sex and diabetes is dependent on the type of diabetes. In our previous study in a leptin receptor deficient model of Type 2 diabetes, contractions of arteries from diabetic female mice were increased to serotonin similar to increases in arteries from diabetic male mice [20]. In Type 2 diabetic mice, female sex did not provide protection from development of vascular dysfunction. However, there was a sex-dependent difference in the mechanisms involved in the dysfunction. There was no increase in protein expression of either rhoA or rho kinase in arteries from either male or female diabetic mice but activation of both rhoA and rho kinase assessed biochemically was increased only in arteries from diabetic males [20]. In the present study our conclusion that activation of rho kinase was increased in a model of Type 1 diabetes is based on the ability of different inhibitors of rho kinase to significantly reduce contractions of aorta to serotonin to levels measured in untreated nondiabetics. In contrast to our previous study in Type 2 diabetics, there was no difference in basal activation of either rhoA or rho kinase in aorta from nondiabetic versus Type 1 diabetic males or females (data not shown). It is possible that differences in activation of rhoA and rho kinase in Type 1 diabetics may only be uncovered following serotonin administration. Our data suggest that in both Type 1 and Type 2 models of diabetes activation of the rhoA/rho kinase pathway may be increased in arteries from diabetic males despite no change in protein expression. These results suggest that other mechanisms regulating activation of rhoA and rho kinase may vary by sex and are altered in diabetes. 

 Differences in glucose and lipid metabolism in Type 1 versus Type 2 diabetes may also contribute to the mechanisms underlying vascular dysfunction. Both forms of diabetes exhibit altered glucose regulation, but hyperlipidemia is most frequently associated only with Type 2 diabetes. Hypercholesterolemia alone increases activation of rho kinase and calcium sensitization of vascular smooth muscle and may contribute to altered vascular function independent of effects of hyperglycemia [34]. Although blood glucose levels were more than doubled in both streptozotocin-treated males and females, the level of hyperglycemia was greater in males. The association between hyperglycemia and vascular disease with worse outcomes has been noted but the relationship between vascular disease and glucose levels above 200 mg/dl has not been tested [35]. It is surprising in the present study that despite glucose levels in females over 400 mg/dl little or no vascular dysfunction was found. Perhaps longer durations of hyperglycemia are needed before protective mechanisms are exhausted in blood vessels from female diabetics. These results also highlight the importance of carefully considering the diabetic model under investigation. Results from each model cannot necessarily be extrapolated to the other.

 Our findings that activity of rhoA and rho kinase is increased in diabetic vasculature in the absence of a change in protein expression highlight the complexity of the mechanisms that regulate rho/rho kinase. Activation of rhoA and downstream rho kinase can be regulated on several levels independent of protein expression. First, inactive rhoA is stored in the cytoplasm bound to GDP. Upon activation, rhoA translocates to the plasma membrane and GTP must be exchanged for GDP. These separate but integrated events are under the control of a family of GTPase regulatory proteins. Rho GTPase regulatory proteins (guanine dissociation inhibitors, guanine exchange factors, and GTPase activating proteins) control the balance between active and inactive rhoA, binding of GDP and GTP, and translocation of rhoA to the plasma membrane. Variants in the gene for rho guanine exchange factor which is involved in the exchange of GTP for GDP are associated with impaired glucose tolerance and Type 2 diabetes [36, 37]. Expression and/or activity of these proteins may be regulated differently in arteries from males compared to females and in nondiabetics compared to diabetics. In addition to rhoA and rho kinase contributing to alterations in contractile dysfunction in diabetes, numerous studies have identified other signaling pathways including protein kinase C contributing to changes in calcium sensitization of contractile proteins in vascular smooth muscle. 

 Another surprising finding in the present study was the effect of chronic treatment with fasudil on vascular function. Studies with chronic treatment with fasudil in animal models of hypertension and myocardial infarction suggest that inhibition of rho kinase decreases blood pressure and improves cardiac function [19, 28, 29]. In an insulin-resistant Type 2 model of diabetes in rats, fasudil prevented the development but could not reverse established metabolic abnormalities and nephropathy [38]. Several mechanisms were proposed to account for the benefits of fasudil treatment. First, insulin signaling in skeletal muscle may be augmented by fasudil to improve glucose metabolism since excess activation of rho disrupted insulin signaling [39, 40]. Second, in mice genetically altered to produce excess rho activation, adipogenesis was inhibited resulting in a reduction in their overall size [39, 40]. It is unlikely that an excess rho activation contributed to the weight loss in our model of diabetes since fasudil treatment had no effect on body weight of diabetic mice. Third, inhibition of rho kinase may inhibit macrophage infiltration, a contributing factor to development of interstitial fibrosis and vascular disease [38]. The failure of fasudil to reverse diabetic nephropathy once it was established was not fully explained [38]. The benefits of fasudil in prevention of diabetic renal dysfunction contrast with the findings in the current study where chronic treatment with fasudil did not prevent development of vascular dysfunction but was associated with augmented contractile responses to serotonin. This effect was specific for serotonin since contractions to thromboxane mimetic U46619 were not affected by chronic treatment with fasudil. These disparate results with fasudil treatment between our study in Type 1 diabetes and the previous work in Type 2 diabetes may relate to differences in target tissue (kidney versus vasculature) or insulin levels. In our model of streptozotocin-induced diabetes, beta cells and insulin levels are decreased; thus, inhibition of rho kinase cannot improve insulin signaling. The diametrically opposed effects of acute versus chronic administration of fasudil in our study highlight the need for continued study of the role of rho kinase in vascular dysfunction in diabetes.

 Although the actions of fasudil, Y27632 and H1152, are ascribed to their ability to inhibit rho kinase, the possibility remains that other unidentified mechanisms are involved. Comparisons of the specificity of these rho kinase inhibitors against several protein kinases suggest that they are all inhibitors of protein kinase A, C, G and even CAM kinase II [21]. Although none are pure rho kinase inhibitors, fasudil is the least and H1152 is the most specific [21]. Clinical trials in the treatment of cerebral vasospasm and angina, however, have not identified serious adverse effects of fasudil beyond an increase in pyrexia [41, 42]. The long-term consequences of inhibitors of rho kinase and their specificity in the treatment of vascular disease in patients with Type 1 versus Type 2 diabetes needs further investigation prior to initiation of their wide spread use in diabetics.

## Figures and Tables

**Table 1:** Body weight and blood glucose levels in nondiabetic and streptozotocin-treated diabetic mice.

	*n*	Weight (g)	Glucose (mg/dl)
Nondiabetic Male	28	27.9 ± 0.4	186 ± 12
Diabetic Male	37	22.6 ± 0.6^†^	510 ± 9^†^
Nondiabetic Female	20	23.0 ± 0.5*	184 ± 12
Diabetic Female	28	19.2 ± 0.5^†^*	434 ± 16^†^*

Mean ± SEM, ^†^ *P* < .05 versus same sex Nondiabetic, and *P < .05 versus Male.

**Table 2:** Body weight and blood glucose levels in nondiabetic and streptozotocin-treated diabetic male mice without and with fasudil treatment (100 mg/kg/day po).

	*n*	Weight (g)	Glucose (mg/dl)
Nondiabetic	8	28.96 ± 1	213 ± 10
Nondiabetic plus Fasudil	8	30.17 ± 1	200 ± 8
Diabetic	10	26.07 ± 1	458 ± 32*
Diabetic plus Fasudil	10	26.04 ± 1	511 ± 11*

Mean ± SEM, *P < .05 versus Nondiabetic.
